# Inflammation Resolution and the Induction of Granulocyte Apoptosis by Cyclin-Dependent Kinase Inhibitor Drugs

**DOI:** 10.3389/fphar.2019.00055

**Published:** 2019-02-19

**Authors:** Jennifer A. Cartwright, Christopher D. Lucas, Adriano G. Rossi

**Affiliations:** ^1^Queen's Medical Research Institute, University of Edinburgh Centre for Inflammation Research, Edinburgh BioQuarter, Edinburgh, United Kingdom; ^2^MRC Centre for Regenerative Medicine, University of Edinburgh, Edinburgh BioQuarter, Edinburgh, United Kingdom

**Keywords:** neutrophil, Mcl-1, R-roscovitine, AT7519, efferocytosis, eosinophil

## Abstract

Inflammation is a necessary dynamic tissue response to injury or infection and it's resolution is essential to return tissue homeostasis and function. Defective or dysregulated inflammation resolution contributes significantly to the pathogenesis of many, often common and challenging to treat human conditions. The transition of inflammation to resolution is an active process, involving the clearance of inflammatory cells (granulocytes), a change of mediators and their receptors, and prevention of further inflammatory cell infiltration. This review focuses on the use of cyclin dependent kinase inhibitor drugs to pharmacologically target this inflammatory resolution switch, specifically through inducing granulocyte apoptosis and phagocytic clearance of apoptotic cells (efferocytosis). The key processes and pathways required for granulocyte apoptosis, recruitment of phagocytes and mechanisms of engulfment are discussed along with the cumulating evidence for cyclin dependent kinase inhibitor drugs as pro-resolution therapeutics.

## Non-Resolving Inflammation

Inflammation is a required dynamic tissue response to injury or infection that is vital for health and, in the majority of cases, will lead to a return of tissue homeostasis (Nathan, 2002; Jones et al., 2016; Robb et al., 2016). All organisms have innate mechanisms for dealing with invading pathogens and tissue damage and these are complemented by adaptive immunity responses in many systems. The responding inflammatory cells and molecular events, which preside after acute tissue damage, act to limit further injury and orchestrate efficient inflammation resolution and restoration of tissue function (Gilroy et al., 2004; Lawrence and Gilroy, 2007; Serhan et al., 2007; Poon et al., 2014). Dysregulated or persistent inflammation following an insult, contributes significantly to the pathogenesis of numerous, often common and difficult to treat conditions in human medicine (Nathan and Ding, 2010; Mantovani et al., 2011; Headland and Norling, 2015; Robb et al., 2016). Examples include atherosclerosis, rheumatoid arthritis, chronic obstructive pulmonary disease, asthma, acute respiratory distress syndrome, Crohn's disease, ulcerative colitis, glomerulonephritis, neurodegenerative disease and multiple sclerosis, with non-resolving inflammation also implicated in both obesity and cancer. Together, these conditions cost the healthcare system in excess of $1 trillion annually in the United States, as well as additional costs in lost productivity within the workplace (Center for Medicare and Medicaid Services, 2016; Buttorff et al., 2017).

Finding approaches to improve inflammation resolution has therefore been an intense area of research, and pharmacologically inducing the clearance of effete inflammatory cells from injured tissue shows promise for future therapeutic application.

This review will cover the mechanisms of inflammation resolution, focusing on granulocyte death and clearance. In particular, how cyclin-dependent kinase inhibitor (CDKI) drugs impact these mechanisms and their use to promote resolution.

## The Transition From the Initiation and Propagation of Inflammation to Its Resolution

To be able to pharmacologically induce or manipulate this transitional stage of inflammation resolution, an understanding of its key processes and pathways is required.

### The Insult and Innate Cellular Response

The primary insult or pathogen is often first recognized by tissue pattern recognition receptors (PRRs) on resident immune cells (including granulocytes, macrophages, dendritic cells, and mast cells), as well as by tissue parenchymal cells (especially epithelial cells). Stimulation of PRRs leads to activation of inflammatory cascades with subsequent release of mediators, including lipids (e.g., leukotriene B_4_, etc.) and small peptides (e.g., interleukin 8, TNF, etc.), which act to attract and/or activate additional immune cells. Neutrophils and eosinophils are cells of the granulocyte lineage that can rapidly migrate and extravasate from the circulation in response to these inflammatory cues. Additional tissue signals also drive granulocyte recruitment through a variety of ligand-receptor interactions, including the FcγR, macrophage-1 antigen, formyl peptide receptor 1 (FPR1) and 2 and the lymphocyte function–associated antigen (Mayadas et al., 2014; Dahlgren et al., 2016). Chemokines, such as CXCL1, CCL2, and CXCL10, are also released from tissues during infections and in sterile injury, by dying cells (Garg et al., 2017) leading to granulocyte recruitment.

These cells will act to contain and kill invading pathogens through phagocytosis, exposure to phagolysosome contents including elastase, myeloperoxidase, reactive oxygen species and proteases (Segal, 2005; Nauseef, 2007; Nauseef and Borregaard, 2014), and through release of extracellular traps (netosis) (Brinkmann et al., 2004; Jorgensen et al., 2017). In the event of sterile inflammation, responding neutrophils will help phagocytose debris but they also have numerous properties that can cause profound local tissue destruction with amplification of inflammation. Neutrophil-derived hydrolytic, oxidative, and pore-forming molecules and certain death pathways, such as necrosis and netosis, have the potential to exacerbate inflammation and tissue injury, resulting in the progression from acute to chronic inflammation and autoimmune conditions (Michlewska et al., 2007; Almyroudis et al., 2013; Tiyerili et al., 2016; Rosales, 2018). It is therefore crucial for granulocytes to be cleared from sites of inflammation and infection once they become effete (Lawrence and Gilroy, 2007; Duffin et al., 2010; Fox et al., 2010).

Far from being a passive process, the resolution of inflammation is an active and highly regulated transition, which is often initiated simultaneously with inflammation onset. It allows a limited effective response, prevents further tissue damage and allows organ repair and functional restoration (Walker et al., 2005). The processes fundamental to inflammation resolution include a change of several mediators and their receptors, for example lysophosphatidylcholine and CX3CL1 released from apoptotic cells or ATP and UTP, which bind to the P2Y2 nucleotide receptor (Lauber et al., 2003; Truman et al., 2008; Elliott et al., 2009). Along with these example changes, the release of lipoxins and annexin 1, which bind to the lipoxin A4 receptor (ALX or FPR2), is another fundamental resolution process that discontinues neutrophil diapedesis (Perretti et al., 2002). Various specialized pro-resolution lipid mediators have been identified, including lipoxins, protectins, and maresins (Buckley et al., 2014). Finally, changes in cellular polarity secondary to the redistribution of organization molecules, such as glycogen synthase kinase-3β, Akt and protein kinase C are important for altering many important biological processes, including chemotaxis. Limiting further tissue neutrophil or eosinophil infiltration is a therapeutic strategy along with inducing processes to remove recruited or expanded inflammatory cell populations (Hallett et al., 2008; Alessandri et al., 2013). Apoptosis of effete inflammatory cells and their effective and timely clearance by surrounding phagocytes is a major process responsible for inflammation resolution (Reville et al., 2006; Serhan et al., 2007; Norling and Perretti, 2013; Poon et al., 2014; Jones et al., 2016; Felton et al., 2018) and this particular process can be enhanced by CDKIs (see below).

### Clearance of Granulocytes

Neutrophils and eosinophils may be eliminated from tissues by several pathways, including reverse migration, lymphatic drainage, exudation to the external environment or local cell death, followed by efferocytosis. There are various forms of local granulocyte death; apoptosis, autophagy, pyroptosis, necrosis, necroptosis and netosis (Heasman et al., 2003; Remijsen et al., 2011; Vanden Berghe et al., 2014) and these can fundamentally influence the inflammatory environment (Almyroudis et al., 2013; Gilroy and De Maeyer, 2015). For example, delayed neutrophil apoptosis occurs in cystic fibrosis, a chronic inflammatory lung disease, characterized by persistent, neutrophil predominant, airway inflammation (McKeon et al., 2008; Moriceau et al., 2010). In this pathological condition, the absence of spontaneous neutrophil apoptosis leads to increased neutrophil death by neutrophil extracellular trap formation (netosis) and worsening inflammation (Gray et al., 2018). Necrosis of inflammatory cells results in membrane integrity loss and release of histotoxic products, such as proteases and reactive oxygen species (Rydell-Törmänen et al., 2006a; Vanden Berghe et al., 2014), whereas apoptosis is generally considered a non-inflammatory form of cell death (McColl et al., 2007). During apoptosis there is cessation of inflammatory cell secretory competence and subsequent recognition by macrophages (Savill and Wyllie, 1989; Whyte et al., 1993; Savill et al., 2002), which is a key element for inflammation resolution (Thieblemont et al., 2018). Neutrophil apoptosis occurs and concludes rapidly (usually < 24 h *ex vivo*) and the process can be difficult to detect in tissue where the apoptosis rate is high, such as in bacterial LPS-induced acute lung injury, as well as in physiological states (McGrath et al., 2011; Lucas et al., 2014). Driving cellular apoptosis has been exploited for therapeutic gain, initially in the treatment of cancers to drive tumor cell death (Green and Walczak, 2013). More recently, driving granulocyte apoptosis with CDKI drugs has successfully improved the resolution of several models of acute and established inflammation (Rossi et al., 2006).

## Apoptosis Pathways

During apoptosis, cellular connections with adjacent cells are lost and there is marked nuclear chromatin and cytoplasmic condensation, resulting in significantly reduced cell size. Chromatin, along with the nucleosome, is cleaved into fragments of approximately 180 base pairs shortly after the initiation of cell death (Wyllie et al., 1984). The plasma membrane undergoes blebbing, invaginations and the generation of fragmentated apoptotic bodies, which have been visualized *in vitro* (Dorward et al., 2014) and *in vivo* (Hochreiter-Hufford et al., 2013). There are also numerous cellular membrane receptor, lipid and protein changes mediating timely phagocytosis and thereby aiding the switch of inflammation to resolution.

Apoptosis, including apoptosis of granulocytes, is an active and tightly regulated form of programmed cell death (Kerr et al., 1972; Jones et al., 2016). CDKIs induce granulocyte apoptosis, which disables the inflammatory cell effector functions, whilst maintaining membrane integrity and thereby avoiding stimulation of the adaptive immune system and maintaining self-tolerance (Duffin et al., 2009; Kushwah and Hu, 2010; Arandjelovic and Ravichandran, 2015). This process is triggered by activation of either of two pathways; the intrinsic pathway, mediated by mitochondria and the extrinsic pathway, mediated by cell surface death receptors. It is now known that there is frequent crosstalk between these pathways (Leitch et al., 2008; Poon et al., 2014), as molecules from one pathway can affect the other (discussed further below) (Li et al., 1998; Igney and Krammer, 2002). Both pathways activate caspases (cysteine aspartyl-specific proteases), as it is the eventual activation of these caspases with subsequent cleavage of cellular substrates, that leads to the biochemical and structural changes of apoptosis (Riley et al., 2006).

### The Intrinsic Pathway

The intrinsic pathway in granulocytes is activated when pro- apoptotic proteins of the Bcl-2 family, including Bax, Bad, Bak and Bid, outweigh the anti-apoptotic Bcl-2 proteins, including myeloid cell leukemia factor-1 (Mcl-1) and B cell lymphoma-extra large (Bcl-XL). The trigger for this includes diverse stimuli including endoplasmic reticulum stress, DNA damage or exposure to pharmacological agents, such as CDKIs.

Neutrophil pro-apoptotic protein expression (Bax, Bad, and Bak) is constitutive (Moulding et al., 2001; Cowburn et al., 2002), whereas pro-survival proteins, or anti-apoptotic Bcl-2 family members (Mcl-1, A1, Bcl-XL) are usually increased or maintained during inflammation secondary to pro-survival mediators (Chuang et al., 1998; Moulding et al., 1998; Fulop et al., 2002).

**Figure 1:**
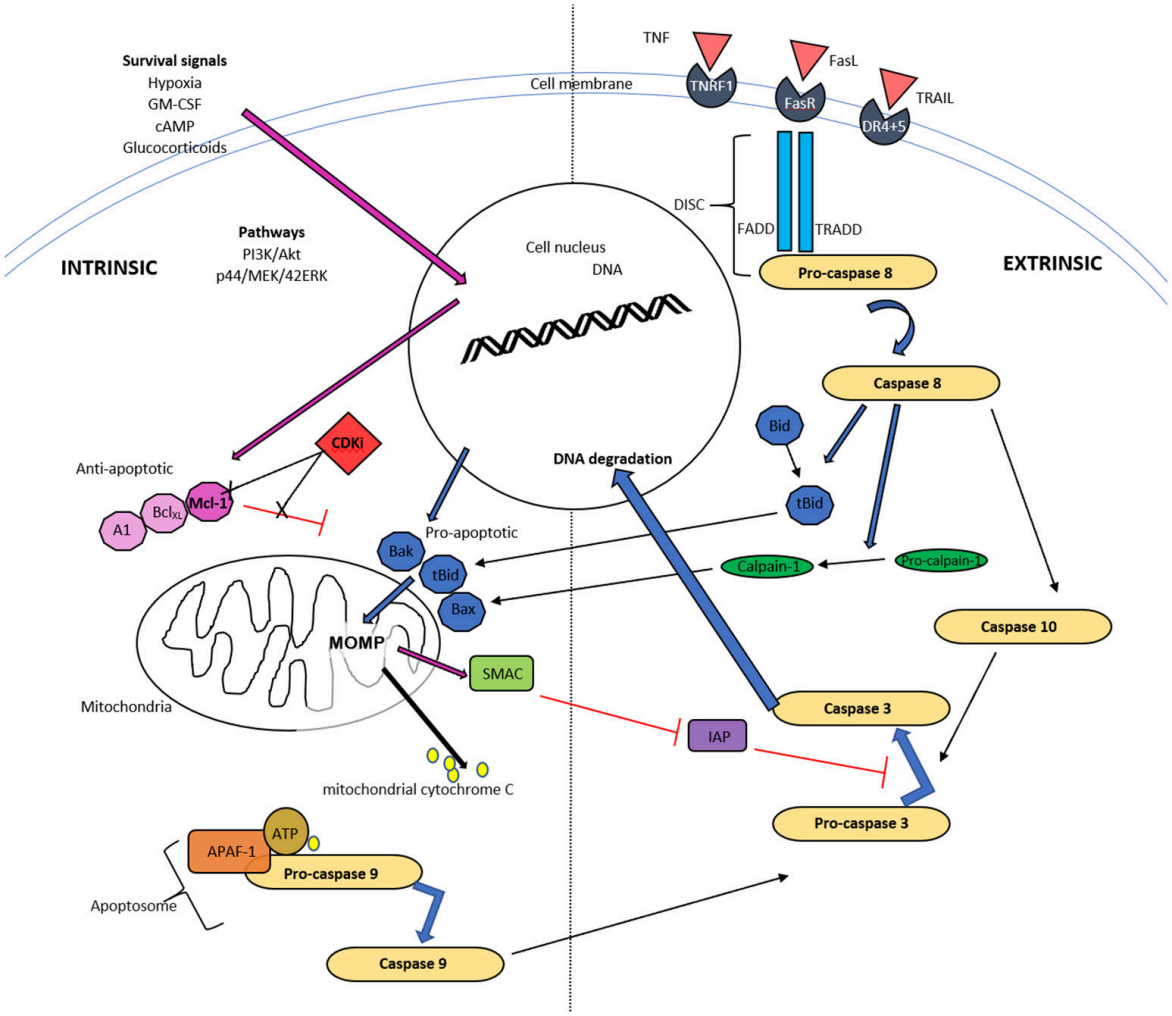
Schematic diagram of intrinsic and extrinsic pathways of neutrophil apoptosis. The intrinsic pathway is instigated when apoptotic proteins outweigh antiapoptotic proteins of the Bcl-2 family and trigger mitochondrial outer membrane permeability (MOMP). The resulting release of cytochrome C, ATP and apoptotic protease activating factor-1 (APAF-1) activates caspase 9 and subsequently caspase 3. Mitochondria also release a second mitochondrial-derived activator of caspases (SMAC), which inhibits the inhibitor of apoptosis (IAP) and thereby enhances apoptosis. Cyclin dependent kinase inhibitors (CDKI) down regulate Mcl-1 of the Bcl-2 proteins, thereby initiating the first step of this pathway. The extrinsic pathway commences with ligation of a death receptor by TNF, Fas ligand or TRAIL. This results in the generation of the death-inducing signaling complex (DISC) cascade and this causes pro-caspase 8 cleavage. Caspase 8 can activate MOMP via cleavage and activation of Bid and can activate caspase 10, and subsequently executioner caspases, typically caspase 3.

A relative reduction of translocated anti-apoptotic proteins to mitochondria, triggers development of mitochondrial outer membrane permeabilisation (MOMP). This allows mitochondrial cytochrome C and other apoptogenic factors to move into the cytosol and bind with APAF1 (apoptotic protease activating factor-1), ATP and the inactive caspase, procaspase-9, together termed the apoptosome. This leads to activation of pro-caspase 9 to caspase 9 (Figure 1). Although neutrophils have low numbers of mitochondria compared to many other cell types, such as hepatocytes, the loss of MOMP is an important and characteristic event of constitutive apoptosis (Maianski et al., 2004; Tait and Green, 2010) and is induced by CDKIs as discussed later. Interestingly, neutrophils have only trace amounts of cytochrome C but this is still necessary for APAF-1–dependent caspase activation (Pryde et al., 2000; Murphy et al., 2003). As well as cytochrome C, mitochondria release SMAC (second mitochondria-derived activator of caspases), which likely has a pro-apoptotic action by inactivating the inhibitor of apoptosis proteins (IAP) (Altznauer et al., 2004). Within neutrophils, Mcl-1 is a key Bcl-2 pro-survival protein instead of Bcl-2 or Bcl-XL (Edwards et al., 2004). In addition, the pro-apoptotic Bcl-2 homologue, Bim, appears to be less important in pharmacologically induced neutrophil apoptosis (Leitch et al., 2010). Mcl-1 can be processed rapidly in the proteasome, which gives it a very short half-life of approximately 2 h (compared to the 12 h half-life of proapoptotic proteins Bax, Bid, and Bim). This short half-life is due to targeted degradation of this protein by the 26S proteasome, secondary to constitutive ubiquitination, and it is also recognized that the PEST domains (proline, glutamic acid, serine and threonine) contribute to this short half-life (Zhong et al., 2005). Neutrophils are therefore exquisitely sensitive to alterations in Mcl-1 with consequent modulation in apoptosis, which likely contributes to the relatively selective apoptosis caused by some CDKIs.

Mcl-1 can also be upregulated or stabilized with various factors to enhance neutrophil survival, such as hypoxia, which delays neutrophil apoptosis by the activation of p38 MAPK signaling and induction of Mcl-1 (Leuenroth et al., 2000). Granulocyte–macrophage colony-stimulating factor (GM-CSF) promotes survival by enhanced Mcl-1 stability through PI3K/Akt and p44/MEK/42ERK signaling pathways (Derouet et al., 2004). Proteasome inhibitors, epoxomicin, lactacystin, bortezomib, or cyclic AMP (cAMP) agonists, dibutyryl cAMP and prostaglandin D_2_ and E_2_, delay neutrophil apoptosis (Rossi et al., 1995; Lucas et al., 2013) and this has been shown to be associated with Mcl-1 stabilization (Kato et al., 2006). Finally, glucocorticoids induce eosinophil apoptosis and enhance their uptake (Meagher et al., 1996; Liu et al., 1999), but their impact on neutrophil apoptosis, which is generally a retardation in apoptosis, is dependent on certain additional environmental factors such as oxygen concentrations and GM-CSF (Marwick et al., 2013).

### The Extrinsic Pathway

The extrinsic “death receptor” pathway is activated by extracellular factors, such as tumor necrosis factor (TNF), tumor necrosis factor-alpha related apoptosis-inducing ligand (TRAIL) and Fas ligand (Soengas et al., 1999), which bind with their cognate receptor, resulting in receptor trimerization. This process causes interactions of “death domains” with the intracellular portions of these receptors, allowing an interaction with several other proteins, such as the FADD adapter protein and clusters of pro-caspase-8 (Ward et al., 1999). These protein communications can lead to induction of the death-inducing signaling complex (DISC) cascade and this results in pro-caspase 8 cleavage. Although mediator binding to TNF receptor, TRAIL receptor and FasLreceptor are described as leading to apoptosis, this is far from the sole action of these receptors and all have been identified as important for many other cellular functions, such as priming and chemotaxis (Anderson et al., 1997; Planells-Ferrer et al., 2016). Caspase 8, as detailed above, can also trigger MOMP via cleavage and therefore activation of one of the pro-apoptotic mediators, Bid (BH3 interaction death agonist) and is one of the main mechanisms of cross talk between the intrinsic and extrinsic pathways (Li et al., 1998, Figure 1).

Activated caspase 8 results in caspase 10 activation and this is where the 2 pathways, intrinsic and extrinsic, converge (Fox et al., 2010). The resulting caspases cleave caspase 3, which is one of the terminal transmitters leading to DNA fragmentation, as well as cleavage of intracellular proteins. Additionally, there are other “executioner caspases,” caspase 6 and caspase 7, which when active will result in a proteolytic deluge of caspase activation (Rathmell and Thompson, 1999).

## Efferocytosis and the Inflammatory Switch

Following the initiation of neutrophil apoptosis, it is imperative that these cells are identified, phagocytosed and cleared from tissue. Apoptosis leads to shutdown of the secretory capacity of granulocytes (Whyte et al., 1993; McColl et al., 2007) but if they are not cleared promptly this will progress to secondary necrosis, with membrane integrity loss and intracellular toxic content release into the surrounding tissue. The recognition and subsequent phagocytosis of apoptotic cells also induces changes in the macrophage, as well as surrounding tissues (Han et al., 2016). Efferocytosis commences the reprogramming of inflammation toward resolution (Savill and Wyllie, 1989; Savill et al., 2002; Poon et al., 2014) and this process can be enhanced by CDKIs (Alessandri et al., 2011).

The phagocytosis of apoptotic cells or cell bodies is mainly completed by professional phagocytes, such as macrophages or dendritic cells. Multiple neighboring cell types can also phagocytose and clear apoptotic cells. Airway epithelial cells phagocytose apoptotic eosinophils and other apoptotic cell bodies but do not appear to phagocytose apoptotic neutrophils (Sexton et al., 2004; Juncadella et al., 2013). Macrophages have been shown to influence efferocytosis by non-professional phagocytes, through the release of insulin-like growth factor 1 (IGF-1) (Han et al., 2016). In this study macrophage IGF-1 dampened the airway epithelial cell engulfment of larger apoptotic cells, while increasing that of microvesicles; preventing IGF-1 signaling resulted in worse inflammation *in vivo*. Retinal pigment epithelial cells are also able to efferocytose and in physiological states engulf photoreceptor outer segments (Finnemann et al., 1997). Hepatocytes can efficiently efferocytose dead cells and this clearance is important both during homeostasis and during inflammatory conditions (Davies et al., 2018). Neutrophils are known to phagocytose cell debri and have additionally been shown to complete efferocytosis of effete neutrophils in a mouse LPS model of pulmonary inflammation by electron microscopy (Rydell-Törmänen et al., 2006b).

The recognition and engulfment of apoptotic cells involves several processes. Firstly, the dying cells release “find me” signals, that mediate phagocyte cellular recruitment and priming, and secondly, the non-viable cells can be recognized by several structural and molecular changes termed “eat me” signals.

### “Find Me” Signals

These are important, particularly when neighboring cells do not have phagocytic potential and tissue resident macrophages or local dendritic cells are required to mediate clearance (Ravichandran, 2003). This may be particularly important in tissues with a relative rarity of resident phagocytes during health, such as the lung, which has less than one alveolar macrophage per 3 alveoli. Similarly, recruitment is important if the tissue injury results in a reduction of tissue resident macrophages, such as Kupffer cells following acute acetaminophen induced injury (Zigmond et al., 2014). The signals identified include lysophosphatidylcholine, which is released from apoptotic cells *in vitro*, via caspase 3 mediated activation of the calcium-independent phospholipase A_2_ (Lauber et al., 2003). A soluble 60kDa fragment of the fractalkine (CX3CL1) protein can serve as a leukocyte find-me signal and has been shown to be released during apoptosis (Truman et al., 2008). Sphingosine 1-phosphate (S1P), a soluble molecule, has also been proposed as a find-me signal. It is released during cell death when sphingosine kinase 2 (SphK2) is cleaved in a caspase-1–dependent manner, and is coupled to phosphatidylserine exposure (Weigert et al., 2010). Finally, nucleotide release from apoptotic cells has also been shown to act as phagocyte chemoattractants. ATP and UTP released from early apoptotic cells can attract monocytes, via the P2Y2 nucleotide receptor *in vitro* and *in vivo* (Elliott et al., 2009). These “find me” molecules attract professional phagocytes and monocytes and are secreted whilst the plasma membrane is still intact and therefore prior to necrosis.

Several of these “find me” signals have yet to be confirmed to be released from granulocytes, though specific neutrophil upregulation of CXCR4 has been implicated in bone marrow macrophage phagocytosis (Furze and Rankin, 2008). It is also possible that “find me” signals are less important for apoptotic granulocyte phagocytosis, given that the inflammatory signals associated with granulocyte infiltration synchronously promote recruitment and expansion of mononuclear phagocytes (Soehnlein et al., 2008).

### “Eat Me” Signals

The structural and molecular changes occurring in apoptotic cells that aid efferocytosis include changes in oxidation state (Slater et al., 1995; Chang et al., 1999), changes in plasma membrane lipid configuration (Kagan et al., 2002), as well as in cell membrane molecule expression (Morris et al., 1984; Homburg et al., 1995). Several of these “eat-me” molecules, which promote engulfment by the phagocyte, have been identified.

One of the key molecules expressed on apoptotic cells, and evolutionary conserved across species, is the lipid phosphatidylserine (Fadok et al., 1992, 1998a; Segawa and Nagata, 2015). Phosphatidylserine (PS) is normally retained on the surface of the plasma membrane through the actions of scramblases and flippases but during apoptosis, scramblase activation and flippase inactivation lead to the appearance of PS on the outer leaflet (Bratton et al., 1997; Schlegel and Williamson, 2001). Phospholipid scrambling is Ca^2+^ dependent and supported by five transmembrane protein 16 (TMEM16) members and Xkr protein members (Suzuki et al., 2010, 2013), with the Xk-family protein Xkr8 mediating PS exposure during apoptosis. PS exposure is required for appropriate uptake of apoptotic cells (Henson et al., 2001) and phagocytes bind to PS via several recognition mechanisms, which can vary dependent on the phagocyte population and context.

**Figure 2:**
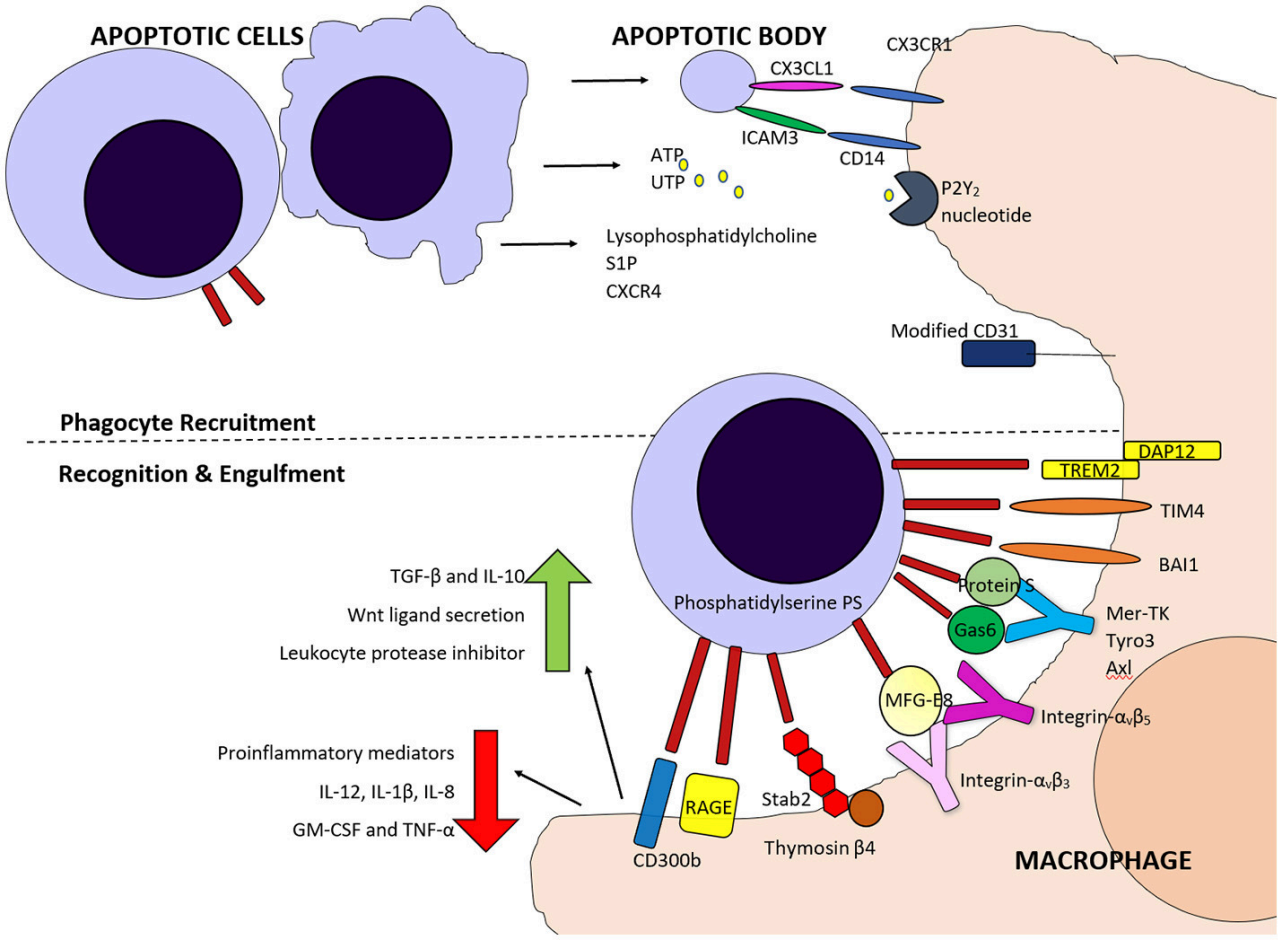
Schematic diagram of some of the molecules involved in phagocyte recruitment and recognition and engulfment of apoptotic cells. Fine me signals including CX3CL1, Sphingosine 1-phosphate (S1P), nucleotides ATP and UTP and lysophosphatidylcholine attract phagocytes, along with inflammatory cytokines. Both direct and indirect binding of phosphatidylserine on apoptotic cells by various molecules is involved in phagocyte detection of these cells and for tethering and engulfment.

PS can be recognized directly by several molecules including; T-cell immunoglobulin mucin protein 4 (TIM4), brain-specific angiogenesis inhibitor 1 (BAI1), Stabilin-2 (Stab2), the receptor for advanced glycation end products (RAGE), triggering receptor expressed on myeloid cells 2 (TREM-2) and CD300B. TIM4 has been identified as a tethering protein which requires cooperation from other molecules for apoptotic cell engulfment to proceed (Dransfield et al., 2015). PS can also be recognized indirectly by use of bridging molecules such as Protein S (Park et al., 2007) and growth arrest-specific 6 (Gas6), (Nakano et al., 1997) which bridge to the Tyro3, Axl, and MerTK (TAM) tyrosine kinase receptors (Rothlin et al., 2015), or MFG-E8, which can bridge to integrins α_v_β_3_ and α_v_β_5_ on phagocytes (Toda et al., 2012) (Figure 2).

Numerous other molecules and receptors have been identified as altered on apoptotic cells. These include CD47, a ligand for signal regulatory protein α (SIRPα), reduced expression of heavily sialylated proteins, calreticulin, annexin I, along with mannose and fucose moieties, which have been reviewed in depth (Barth et al., 2017). It is unknown why so many “find me” and “eat me” signals are present, but the high number identified could indicate a redundancy in the system or, more likely, that molecules provide tissue-specific, cell-specific, context and time dependent control. Some viable cells have been shown to express low levels of PS (Barth et al., 2018), so it is not surprising that additional molecules on viable cells have been defined, that distinguish them from dying cells, so called “don't eat me” signals. CD31 (also known as platelet endothelial cell adhesion molecule-1, PECAM-1) was identified as preventing phagocytosis by human monocyte-derived macrophages *in vitro* (Brown et al., 2002).

## The Anti-Inflammatory Benefit of Apoptosis and Efferocytosis

Clearance of apoptotic cells is crucial to maintain cellular function under physiological and pathological conditions (Michlewska et al., 2007) and is usually an immunologically silent process, as it does not induce immune cell infiltration (Fadok et al., 1998a; Ravichandran and Lorenz, 2007; Arandjelovic and Ravichandran, 2015). The production of find me and eat me signals not only promotes phagocyte recruitment but can activate them and improve phagocytic capacity, for example through upregulation of the bridging molecule MFG-E8 in macrophages, which facilitates engulfment (Miksa et al., 2007). These authors identified that CX3CL1 induces MFG-E8 in macrophages *in vitro* and promotes phagocytosis of apoptotic cells. Endogenous CX3CL1 falls during a rat model of sepsis and is associated with lower MFG-E8. The authors found MFG-E8 can be rescued by injecting CX3CL1, which also improves the sepsis.

Interaction of apoptotic cells with macrophages transforms the macrophage secretory profile by increasing production of anti-inflammatory signals (e.g., TGF-β and IL-10) (Voll et al., 1997; Huynh et al., 2002), and reducing proinflammatory mediators (e.g., IL-12, IL-1β, IL-8, GM-CSF, and TNF) (Fadok et al., 1998b; Filardy et al., 2010). This change in cytokines is likely to have subsequent effects on adaptive immunity as interferon-γ (IFN-γ), which is required for cell mediated immune responses, is induced by IL-12 but inhibited by IL-10 (Paul and Seder, 1994). Interestingly, the interaction of PS on viable monocytes, through the protein S and Mer/Tyro3 receptor tyrosine kinase axis, has been shown to augment inflammatory responses, indicating a duality of function dependent on the type of cell presented (Barth et al., 2018).

The upregulation of alveolar macrophage PPARγ expression and consequent proresolving cytokines has been shown in an acute pulmonary inflammation model after instillation of apoptotic leukocytes (Yoon et al., 2015). Tyrosine receptor kinase Mer, has been shown to be a crucially expressed protein for alveolar macrophage phagocytosis of apoptotic eosinophils, and without this binding mechanism, increased inflammation, delayed resolution and increased airway responsiveness occur (Felton et al., 2018). Phagocytosis of damaged cells by macrophages also prompts Wnt ligand secretion and promotes liver regeneration *in vivo* (Boulter et al., 2012). Murine macrophages have also been found to produce leukocyte protease inhibitor through recognition and removal of apoptotic cells, which could help to attenuate inflammation (Odaka et al., 2003).

Overall the efferocytosis-induced substitution of proinflammatory with anti-inflammatory mediators aids inflammation resolution and permits tissue repair and regeneration. Improved tissue inflammation has been experimentally shown with enhancement of apoptotic cell clearance in acute pulmonary injury (Moon et al., 2010) and by induction of the STAT3–IL-10–IL-6 axis, a positive regulator of macrophage efferocytosis, following hepatic injury (Campana et al., 2018).

Apoptotic neutrophils also have additional beneficial effects in inflammation. In a murine model of septic shock, administered apoptotic neutrophils resulted in reduced proinflammatory cytokines, suppression of further neutrophil infiltration and decreased serum lipopolysaccharide which led to improved survival (Ren et al., 2008). Inducing eosinophil apoptosis has also been shown to attenuate airway inflammation in an ovalbumin mouse model of allergic airway disease (Lucas et al., 2015). Eosinophil apoptosis was induced by wogonin, a flavone, via activation of caspase-3 and another study showed granulocyte apoptosis was secondary to Mcl-1 downregulation, similar to CDKI action (Lucas et al., 2013). Apoptotic granulocytes, specifically neutrophils, have also been shown to secrete lactoferrin which prevents recruitment of additional granulocytes (Bournazou et al., 2009) thereby further mediating inflammation resolution. Even prior to apoptosis, neutrophils supress proinflammatory cytokine secretions *in vitro* from monocyte-derived macrophages via sustained NFκB suppression (Marwick et al., 2018).

## Pharmacological Mechanism of CDKIs

Given that apoptosis and phagocytic clearance of apoptotic granulocytes is key for the resolution of inflammation, it is not surprising that the mechanisms involved have been intensely investigated. One pharmacological approach, targeting this resolution switch, is the use of cyclin-dependent kinase inhibitor drugs, which has been a focus of our laboratory for several years.

Differences in the regulation of apoptotic pathways are present across cell types; these differences can allow for selective targeting, which is appealing where resolving inflammation is required alongside repair and regeneration of local tissue. Several investigations have revealed pharmacological inhibition of cyclin-dependent kinases (CDKs) can induce selective apoptosis of granulocytes with overall positive effects for the injured tissue.

CDKs are present in all known eukaryotes and they have an evolutionarily conserved regulatory function on the cell cycle. CDKs 1, 2, 3, and 4, are directly involved in cell cycle regulation where they complex with their associated cyclin partners during the progression of cell division, through the four phases [G1, S, G2, M) (Vermeulen et al., 2003). Cells contain endogenous CDKI proteins (e.g., p21 variant (v)1, p27Kip1 and cyclin-dependent kinase inhibitor 1C (p57, Kip2)] so the cell cycling process is tightly regulated. Aberrant expression or dysregulation of endogenous CDKs have been identified in several types of cancer development and progression (Johnson and Shapiro, 2010). Therefore, multiple inhibitors (CDKIs) have been developed as potential cancer treatments (Bilgin et al., 2017), with some of these having reached clinical trials (Chen et al., 2014).

Since the discovery that neutrophils and, subsequently, eosinophils, could be driven into apoptosis by CDKIs (Rossi et al., 2006) this has led to the investigation of their use in several inflammatory conditions (Rossi et al., 2007). Neutrophils are terminally differentiated cells and therefore the action of CDKIs on their lifespan was initially surprising, especially as some CDKs (CDK2, CDK4, and CDK6) become downregulated during myeloid cell maturation (Klausen et al., 2004). Additionally, when CDKIs were used on neurons, another terminally differentiated cell type, there was the opposite effect of prolonged cellular survival (Dhavan and Tsai, 2001) while CDKI treatment of cardiomyocytes inhibited doxorubicin-induced apoptosis via reduced CDK2-dependent expression of Bim (Xia et al., 2018).

It is clear from several studies that CDKIs result in neutrophil apoptosis through down-regulation of Mcl-1, which is a key protein regulating neutrophil apoptosis (Moulding et al., 1998; Edwards et al., 2004; Michels et al., 2005). It has been demonstrated that human eosinophils have CDK1,−2,−5,−7, and−9 (Farahi et al., 2011) and neutrophils have CDK 1, 2, 4, 5, 6, 7, and 9 to various levels (Klausen et al., 2004; Rossi et al., 2006; Leitch et al., 2012; Wang et al., 2012) with phosphorylation of RNA polymerase II by CDKs 7 and 9 inhibited by the CDKI R-roscovitine. CDK9 has been shown to be a key regulator of human neutrophil lifespan and it's decline is associated with spontaneous apoptosis (Wang et al., 2012). CDK9 has also recently been shown to be the key CDK governing neutrophil apoptosis and inflammation resolution in zebrafish (Hoodless et al., 2016). This CDK is a component of the positive transcription elongation factor complex, involved in transcriptional regulation (Fu et al., 1999), which is required for maintaining levels of short lived anti-apoptotic proteins, such as Mcl-1. Neutrophil Mcl-1expression has been shown to decline after CDK inhibition resulting in apoptosis, even in the face of various neutrophil pro-survival factors, such as GM-CSF (Kobayashi et al., 2005). The CDKI, purvalanol A, also induces neutrophil apoptosis *in vitro* with an associated increase in the turnover rate of Mcl-1 (Phoomvuthisarn et al., 2018). CDKI induced granulocyte apoptosis is also caspase dependent, as it can be abolished by broad spectrum inhibition of caspases (Rossi et al., 2006; Duffin et al., 2009; Leitch et al., 2010) confirming that CDKIs act upstream of caspase activation. R-roscovitine was shown to cause a significant induction of neutrophil MOMP, alongside this reduction in Mcl-1 (Leitch et al., 2012), consistent with induction of the intrinsic apoptosis pathway. Mcl-1 was also shown to be reduced in mouse derived HoxB8 cells during apoptosis when cultured with R-roscovitine (Gautam et al., 2013).

## Pro-Resolution Effects of CDKIs

Inducing neutrophil apoptosis with CDKIs has been shown to considerably improve the resolution of inflammation in diverse models. Initial studies used the CDKI, R-roscovitine, also known as seliciclib or CYC202, which inhibits multiple enzyme targets including CDK2, CDK7 and CDK9, and is being trailed for various viral, neoplastic and inflammatory conditions (Diwan et al., 2004; Sadaie et al., 2004; Raje et al., 2005; Leitch et al., 2009; Meijer et al., 2016, see Table 1). R-roscovitine was shown to rapidly and efficiently induce caspase-dependent eosinophil apoptosis *in vitro*, which was associated with down regulation of Mcl-1 and induction of MOMP (Duffin et al., 2009, Figure 1). R-roscovitine was also demonstrated *in vitro* to override granulocyte pro-survival mediators, TNF and LPS, to induce neutrophil apoptosis (Leitch et al., 2010). Further insight into CDKI mechanism was also identified in this study, as NF-κB activation and ERK activation were not directly affected during the stimulated apoptosis, but again Mcl-1 was downregulated. Further to the *in vitro* effects of R-roscovitine, this drug was shown to enhance inflammation resolution in mouse models of carrageenan-induced acute pleurisy, bleomycin-induced lung inflammation and serum-induced arthritis (Rossi et al., 2006; Leitch et al., 2008). Human eosinophils express five known targets for R-roscovitine: CDK1,−2,−5,−7, and−9, and this drug induced eosinophil apoptosis and overcame the anti-apoptotic signals from GM-CSF and IL-5 *in vitro* (Farahi et al., 2011). R-rocovitine, used in an ova mouse model of allergic airway disease, resulted in apoptosis of peripheral blood and spleen-derived eosinophils and although this drug did not modulate the pulmonary eosinophilia (Farahi et al., 2011), a later developed CDKI has proved successful (Alessandri et al., 2011; Felton et al., 2014). Neutrophil apoptosis was also induced by R-roscovitine in a zebrafish tail injury model, reducing inflammatory cell numbers (Loynes et al., 2010). R-roscovitine has also been shown to reduce glial activation, neuronal loss and neurological deficits after brain trauma (Hilton et al., 2008) and be neuroprotective in a model of stroke (Menn et al., 2010). In a mouse model of systemic lupus R-roscovitine was associated with extended mouse lifespan, reduced glomerulonephritis with diminished proteinuria and renal damage (Zoja et al., 2007). R-roscovitine has also been shown to have beneficial effects by blocking leukocyte extravasation through inhibition of CDKs 5 and 9 (Berberich et al., 2011). Additional pro-resolution effects of R-roscovitine may also be possible when combining this compound with nitric oxide, as these hybrid compounds have increased pro-apoptotic activity (Montanaro et al., 2013).

**Table 1:** A comprehensive literature summary of the main actions of CDKI drugs on inflammatory cells or inflammatory conditions/models.

**CDKI**	**Main findings and action**	**References**	**Species**	***In vitro, in vivo***	**Organ or cell type**
R-roscovitine 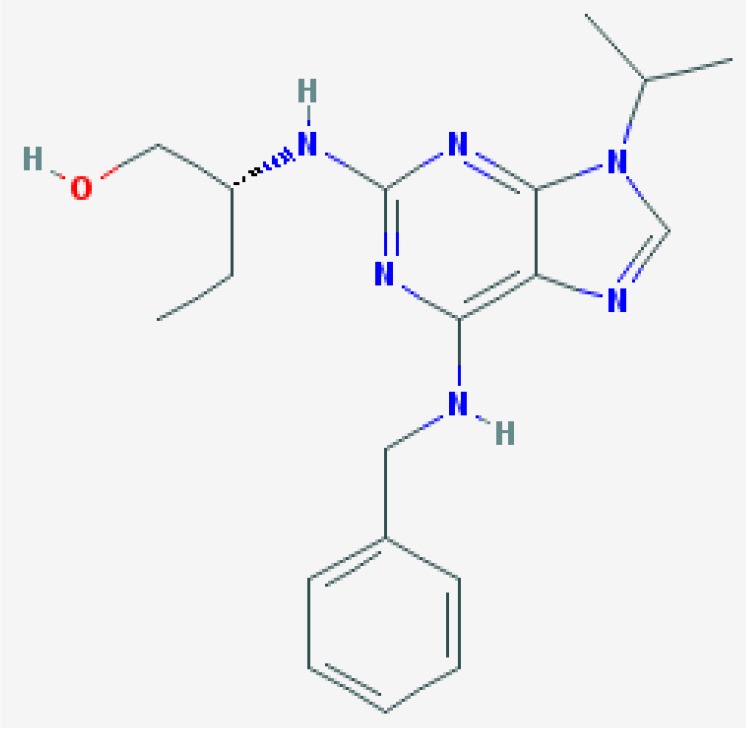 https://pubchem.ncbi.nlm.nih.gov/compound/160355#section=2D-Structure Molecular Weight: 354.458 g/mol Half Life: 30 min, rat (Vita et al., 2005) 1.19 h, mice (Nutley et al., 2005) 2–5 h, man (Benson et al., 2006)	Induced caspase-dependent neutrophil apoptosis with a corresponding down regulation of Mcl-1. Overcame anti-apoptosis signals from GM-CSF and LPS.	Rossi et al., 2006	Human primary cells	*In vitro*	Neutrophils
	Enhance inflammation resolution in mouse models of carrageenan-induced acute pleurisy, bleomycin-induced lung inflammation and serum-induced arthritis, with a decrease in inflammatory cells and edema formation.		Mouse	*In vivo*	Lung Joint
	Reduced *ex vivo* T cell and B cell proliferative responses. Reduced T-cell production of interferon-γ and IL-10 and B cell release of IgG2a.	Zoja et al., 2007	Mouse primary cells	*In vitro*	T and B cells
	Extended mouse lifespan and reduced glomerulonephritis in a model of systemic lupus.		Mouse	*In vivo*	Kidney
	Caspase-dependent eosinophil apoptosis, reduced Mcl-1 and induction of MOMP.	Duffin et al., 2009	Human primary cells	*In vitro*	Eosinophils
	Accelerated recovery from Streptococcus pneumoniae bacterial meningitis, through neutrophil apoptosis. Reduced haemorrhagic events and bacterial titres.	Koedel et al., 2009	Mouse	*In vivo*	CNS
	Over-rides TNF-α and LPS-induced survival to induce neutrophil apoptosis via Mcl-1 reduction.	Leitch et al., 2010	Human primary cells	*In vitro*	Neutrophils
	Reversed delayed apoptosis of neutrophils from patients with cystic fibrosis (CF).	Moriceau et al., 2010	Human primary cells (CF)	*In vitro*	Neutrophils
	Induced neutrophil apoptosis in a zebrafish tail injury model, reducing inflammatory cell numbers.	Loynes et al., 2010	Zebrafish	*In vivo*	Tail fin
	**[Roscovitine (Unspecified which isomer of Roscovitine)]** Inhibited granulocyte TNF-α-evoked expression of endothelial adhesion molecules and inhibited protein kinase A, ribosomal S6 kinase and CDKs 2, 5, 7, and 9.	Berberich et al., 2011	Human primary cells	*In vitro*	Granulocytes
	Decreased TNF-α-evoked leukocyte adhesion and transmigration via cremaster intravital imaging.		Mouse	*In vivo*	Muscle
	Induced eosinophil apoptosis and overcame the anti-apoptotic signals from GM-CSF and IL-5. Enhanced phagocytic clearance of eosinophils by macrophages.	Farahi et al., 2011	Human primary cells	*In vitro*	Eosinophils
	Resulted in apoptosis of peripheral blood and spleen-derived eosinophils in an ova model of allergic airway disease.		Mouse	*In vivo*	Lung, Systemic
	Induced neutrophil apoptosis and MOMP, with CDK 7 and 9 inhibition of RNA polymerase II. Gene expression of pro-survival Bcl-2 homologs were unaffected apart from Mcl-1, which was significantly downregulated.	Leitch et al., 2012	Human primary cells	*In vitro*	Neutrophils
	Propelled resolution of inflammation in the bleomycin-induced lung injury model.		Mouse	*In vivo*	Lung
	Enhanced apoptosis in neutrophils and reduced TNF-α and keratinocyte chemoattractant production in MH-S (alveolar macrophage) and MLE-12/ MLE-15 (respiratory epithelial) cell lines.	Hoogendijk and Roelofs, 2012	Human primary cells. Mouse cell line	*In vitro*	Neutrophils Pulmonary epithelial cells
	Reduced neutrophil numbers in bronchoalveolar lavage fluid during lipoteichoic acid -induced lung inflammation and bacterial burden in an S. pneumoniae infection. There was also a time dependent transient increase in bacterial load.		Mouse	*In vivo*	Lung
	Induced neutrophil apoptosis.	Wang et al., 2012	Human primary cells	*In vitro*	Neutrophils
	Bcl-2 strongly protected against roscovitine-induced apoptosis in neutrophils. Loss of Mcl-1 during apoptosis through post-transcriptional regulatory mechanisms.	Gautam et al., 2013	Mouse primary cells	*In vitro*	Hoxb8 neutrophils progenitors
	Inhibit human blood eosinophil exocytosis through CDK5 inhibition and significantly inhibits degranulation.	Odemuyiwa et al., 2015	Human primary cells and cell lines	*In vitro*	Eosinophils Eosinophil differentiated HL-60
R-roscovitine and nitric oxide hybrid compounds	Both compound 9a and 9c increased pro-apoptotic neutrophil activity.	Montanaro et al., 2013	Human primary cells	*In vitro*	Neutrophils
S-roscovitine	Neuroprotective in a dose-dependent manner in two models of focal ischemia. Resulted in less neutrophils in BAL with no detrimental effect on macrophage numbers.	Menn et al., 2010	Mouse	*In vivo*	Lung
AT7519 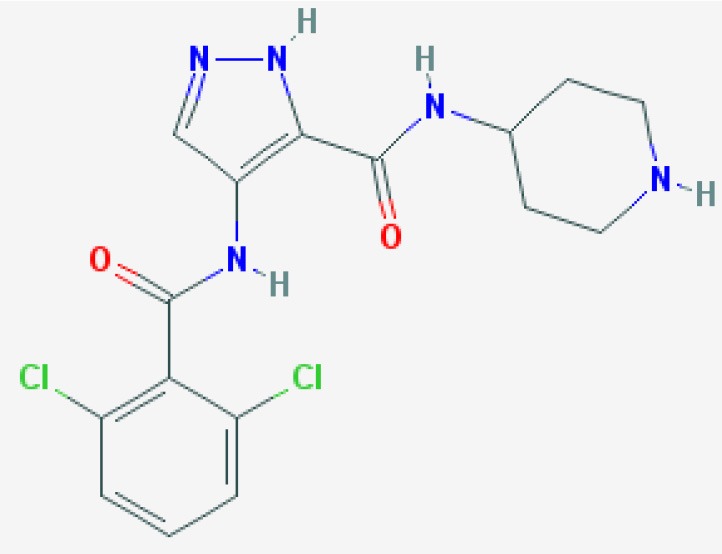 https://pubchem.ncbi.nlm.nih.gov/compound/11338033#section=2D-Structure Molecular Weight: 382.245 g/mol Half Life: 1.8 h, mice (Dolman et al., 2015) 7–10 h, man (Mahadevan et al., 2011)	Induced apoptosis in primary human eosinophils in a concentration dependent manner.	Alessandri et al., 2011	Human primary cells	*In vitro*	Eosinophils
	Resolution of allergic pleurisy by caspase-dependent eosinophil apoptosis and enhanced macrophage ingestion of apoptotic eosinophils.		Mouse	*In vivo*	Lung
	Induced concentration, time, and caspase dependent human neutrophil apoptosis via Mcl-1 downregulation and over came LPS induced survival and lipoteichoic acid and peptidoglycan (PepG). Did not cause human macrophage apoptosis despite downregulation of Mcl-1.	Lucas et al., 2014	Human primary cells	*In vitro*	Neutrophils
	Induced caspase-dependent apoptosis and down-regulates the key survival protein Mcl-1 in mouse bone marrow-derived neutrophils.		Mouse		Neutrophils bone marrow derived
	Improved LPS-induced pulmonary inflammation resolution, neutrophil apoptosis and downregulation of Mcl-1. Enhanced bacterial clearance in established *E. coli* pneumonia and accelerated resolution of infection-associated inflammation. Also accelerated resolution of established lipoteichoic acid/peptidoglycan mediated lung inflammation.		Mouse	*In vivo*	Lung
	Inhibited human blood eosinophil exocytosis through CDK5 inhibition and significantly inhibited degranulation.	Odemuyiwa et al., 2015	Human primary cells and cell line	*In vitro*	Eosinophils Eosinophil differentiated HL-60 cells
	Accelerated resolution of neutrophilic inflammation at the wound site at 24 h post tail fin wounding by induction of neutrophil apoptosis with no reduction in recruitment or reduction in macrophage recruitment.	Hoodless et al., 2016	Zebrafish	*In vivo*	Tail Fin
	Over came ARDS neutrophil prolonged survival to induce apoptosis with reduced expression of the pro-survival protein Mcl-1.	Dorward et al., 2017	Human primary cells (ARDS patients)	*In vitro*	Neutrophils
	Induced neutrophil apoptosis in control and CF neutrophils, correcting the delayed apoptosis in CF to that of control levels. Also reduced neutrophil extracellular trap formation from patients with CF.	Gray et al., 2018	Human primary cells (CF patients)	*In vitro*	Neutrophils
NG75 (Gray et al., 1998; Chang et al., 1999)	Markedly increased neutrophil apoptosis.	Rossi et al., 2006	Human primary cells	*In vitro*	Neutrophils
Purvalanol A 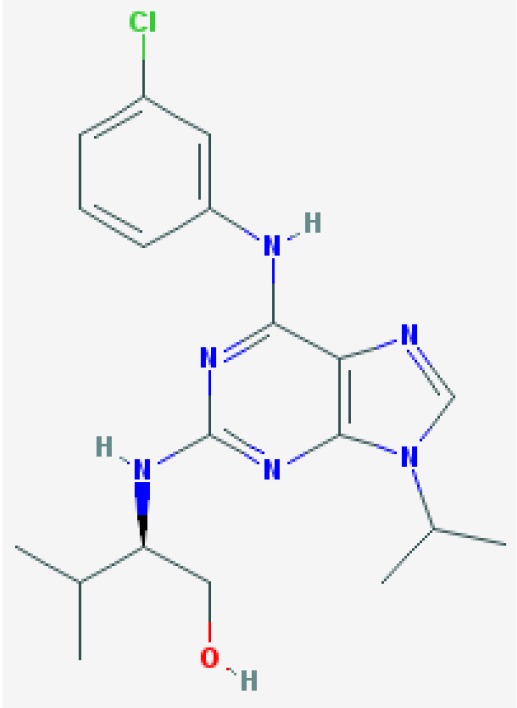 https://pubchem.ncbi.nlm.nih.gov/compound/456214#section=2D-Structure Molecular Weight: 388.9 g/mol	Induced neutrophil apoptosis with increased Mcl-1 turnover and activation of p38-mitogen-activated protein kinase.	Phoomvuthisarn et al., 2018	Human primary cells	*In vitro*	Neutrophils
Flavopiridol 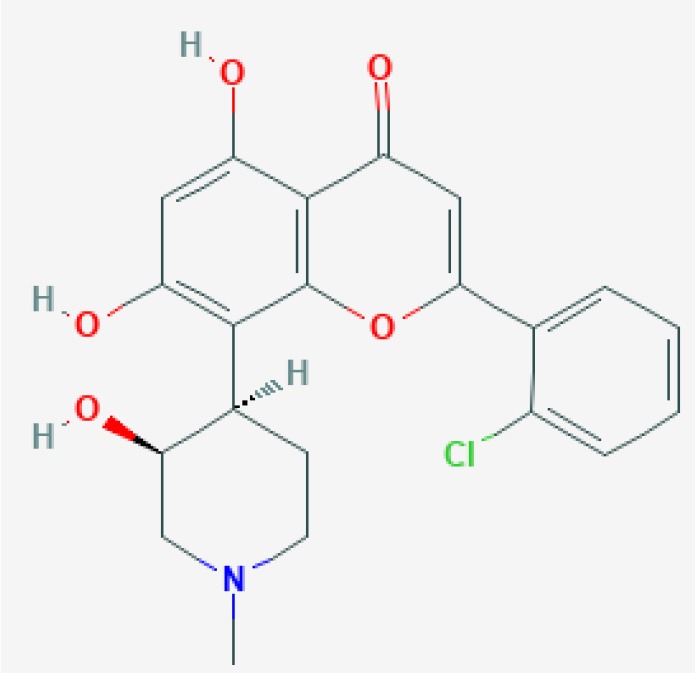 https://pubchem.ncbi.nlm.nih.gov/compound/5287969#section=2D-Structure Molecular Weight: 401.843 g/mol	Increased neutrophil apoptosis and declined Mcl-1. Accelerated resolution of neutrophilic inflammation at the wound site at 24 h post tail fin wounding.	Wang et al., 2012 Hoodless et al., 2016	Human primary cells Zebrafish	*In vitro In vivo*	Neutrophils Tail fin
DRB (5,6-dichloro-1-beta-D-ribofuranosylbenzimidazole) 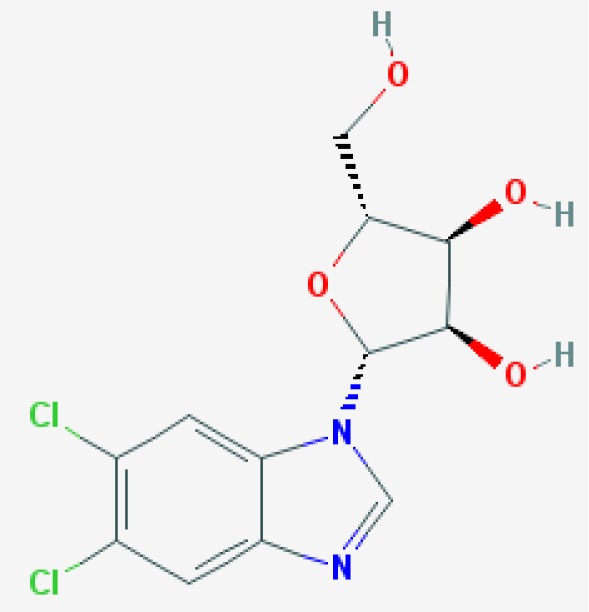 https://pubchem.ncbi.nlm.nih.gov/compound/5894#section=2D-Structure Molecular weight: 319.138 g/mol	Neutrophil apoptosis via specific CDK7 and 9 inhibition	Leitch et al., 2012	Human primary cells	*In vitro*	Neutrophils
	Resolved inflammation in the bleomycin-induced lung injury model. Reduced neutrophils in BAL with no detrimental effect on macrophage numbers.	Leitch et al., 2012	Mouse	*In vivo*	Lung
**Non Inflammatory Cells**					
R-roscovitine	Inhibited CDK2-dependent S-phase re-entry and protected against cardiomyocyte doxorubicin-induced apoptosis.	Xia et al., 2018	Neonatal rat cardiomyocytes and Human cell line	*In vitro*	Cardiomyocytes H9c2
Roscovitine (And CDK1- and CDK4-selective inhibitors).	Attenuated neuronal cell death, decreased microglial activation and microglial-dependent neurotoxicity in primary cortical microglia and neuronal cultures.	Hilton et al., 2008	Rat	*In vitro*	Cell cultures: cortical microglia and neuronal cells
	Decreased brain lesion volume by 37%. Reduced glial activation, neuronal loss and neurological deficits after traumatic brain injury.		Rat	*In vivo*	Brain
Purvalanol A	Attenuated neuronal cell death.		Rat	*In vitro*	Cortical microglia
Flavopiridol	Inhibited CDK9, protected human primary chondrocytes and cartilage explants from the catabolic effects of proinflammatory cytokines.	Yik et al., 2014	Human primary cells and explants	*In vitro*	Chondrocytes Cartilage explants

*Additional relevant, non-chemotherapy related actions of CDKIs are also detailed*.

As granulocytes are crucial to combat infectious diseases, it is also important to know that inducing apoptosis in the face of infection is still beneficial. R-roscovitine, used in wild type mice treated with antibiotics for bacterial meningitis, significantly accelerated recovery by inducing neutrophil apoptosis. In this elegant study, both the number and extent of measurable hemorrhagic events and bacterial titres was reduced by the CDKI (Koedel et al., 2009). This investigation also confirmed that preventing neutrophil apoptosis by genetic upregulation of Bcl-2, resulted in enhanced tissue damage and exacerbation of meningitis. Positive effects of CDKI have also been identified in other models of infectious conditions (Lucas et al., 2014; Dorward et al., 2017). Lucas et al showed a CDKI, AT7519, accelerated bacterial clearance in an established *E. coli* pneumonia model and this effect on bacterial burden was shown to not be direct, but likely secondary to neutrophil apoptosis and cellular clearance. Similarly, Roscovitine added to the antibiotic ceftriaxone has been shown to reduce later bacterial burden (but enhanced early bacterial burden) in an S. pneumoniae model of lung infection, suggesting that the timing of CDKI in infectious disease may be critical (Hoogendijk and Roelofs, 2012).

The more recently developed CDKI, AT7519, is a competitive inhibitor of CDK5 and CDK9, (both kinases are present in human neutrophils) and is in clinical trials for advanced refractory solid tumors or non-Hodgkin's lymphoma (Leitch et al., 2012; Chen et al., 2014). AT7519 has also been shown to improve LPS-induced pulmonary inflammation resolution through downregulation of Mcl-1 (Lucas et al., 2014). Bronchoalveolar and pulmonary neutrophils were both significantly reduced by this CDKI via induction of apoptosis and there was improved alveolar–capillary barrier integrity, measured by BAL IgM. AT7519-induced downregulation of Mcl-1 was also demonstrated in this study to be critical for enhanced inflammation resolution *in vivo*, as blocking the Mcl-1 downregulation with bortezomib (a proteasomal inhibitor) abrogated the beneficial effect on pulmonary inflammation. AT7519 also induces concentration-dependent apoptosis of human eosinophils *in vitro* with 50 times greater potency than R-roscovitine (Alessandri et al., 2011). This CDKI, administered systemically at the peak of pleural inflammation in a eosinophil-dominant pleurisy model, augmented the resolution of inflammation (Alessandri et al., 2011).

AT7519 has been shown to override the complex neutrophil pro-survival environment from systemic sepsis induced acute respiratory distress syndrome (ARDS). Neutrophils isolated from ARDS patients had reduced apoptosis in line with other studies. However, this could be overcome by treatment with AT7519, which also led to Mcl-1 loss and caspase activation in cells from this patient cohort (Dorward et al., 2017). Neutrophils with delayed apoptosis from patients with cystic fibrosis, when cultured with AT7519, showed increased apoptosis and a resulting reduction of extracellular trap formation (Gray et al., 2018). Roscovitine also reversed this delayed apoptosis of cystic fibrosis neutrophils *in vitro* (Moriceau et al., 2010). AT7519 has also been shown to enhance inflammation resolution by neutrophil apoptosis in a zebrafish model of tail fin injury and this process was dependent on CDK9 reduction (Hoodless et al., 2016). Finally, CDK9 inhibition by flavopiridol has also been shown to protect human primary chondrocytes and cartilage explants from the catabolic effects of proinflammatory cytokines (Yik et al., 2014).

## CDKI Impact on Efferocytosis and Resolving Inflammation

It is crucial that efferocytosis continues after treatment with CDKI, especially as there are increased number of apoptotic neutrophils and progression to necrosis will occur if these effete cells are not engulfed. AT7519, used at concentrations that induce neutrophil apoptosis, does not result in monocyte-derived macrophage apoptosis *in vitro*, though does cause a concentration dependent reduction of Mcl-1. Importantly, AT7519 did not reduce the percentage of phagocyting macrophages *in vitro* (Lucas et al., 2014) and in another study, R-roscovitine was shown to enhance phagocytic clearance of eosinophils by macrophages (Farahi et al., 2011). The efficiency of efferocytosis was also shown to be improved in the LPS and pneumonia models in this same study, and in a pleurisy mouse model (Alessandri et al., 2011), an effect likely dependent on enhanced granulocyte apoptosis. Many inflammatory conditions have defects in macrophage phagocytosis or a saturation of this process (Schrijvers et al., 2005; Morimoto et al., 2012; Barnawi et al., 2017; Bukong et al., 2018). Therefore, the effects of additional granulocyte apoptosis, or direct effects of CDKIs in these conditions, need to be investigated and carefully counterbalanced with available engulfment capacity. If not, there is the potential for additional progression to necrosis with possible detrimental consequences. However, whether necrosis of apoptotic neutrophils (secondary necrosis) is as inflammatory as primary necrosis is relatively understudied, but LL-37-induced neutrophil secondary necrosis was shown not to be proinflammatory to macrophages (Li et al., 2009). Saturation of efferocytosis can also provide further anti-inflammatory macrophage phenotypes, such as the pro-resolving, CD11b^low^ subtype identified during the resolution of murine peritonitis (Schif-Zuck et al., 2011), but this effect of CDKIs remains unexplored *in vivo*.

Selective and low doses of CDKIs may impact many other cell types, including those in the surrounding tissues after injury. Whether CDKIs, by effects on cell cycle progression, also interfere with the cellular proliferation and subsequent tissue repair after injury, is an area for further work (Rossi et al., 2006; Alessandri et al., 2011; Lucas et al., 2014).

## Future Studies With CDKIs and Complication Considerations

CDKIs have been utilized in clinical trials to treat human cancer but have not yet been fully trialed in human inflammatory conditions, other than the treatment of patient cells *ex vivo* (Dorward et al., 2017). Given that certain CDKIs have been approved for human trials and their side effect profiles are known, it is not inconceivable to consider these as future potential therapeutic avenues for non-resolving inflammation or to expedite recovery of acute inflammatory conditions. There are still several unexplored inflammatory conditions, occurring in various organs/systems, including chronic disorders, where manipulation of granulocyte apoptosis may have beneficial effects. Ensuring a selectivity for granulocyte apoptosis in these established inflammation models, where there is pre-existing damaged tissue, will also be important. Therefore, work is indicated to establish the breadth of CDKI granulocyte manipulation that is both possible and beneficial.

There are also other inflammation resolving actions of CDKIs that could be explored further. These include phagolysosome acidification in alveolar macrophage from patients with cystic fibrosis, lymphocyte modification and potential analgesic properties, which are reviewed in detail by Meijer et al. (2016).

Lastly, an area remaining unexplored with regards to CDKI, is the potential to pharmacologically harness multiple pathways of the inflammation resolving switch synergistically. For example, it has been shown that proresolving lipids can improved phagocytosis, and an additional form of neutrophil apoptosis, so called “phagocytosis-induced apoptosis” (Serhan et al., 2007; Kebir et al., 2012). Glucocorticoids have also been shown to increase macrophage efferocytosis of apoptotic neutrophils, and *in vitro* and this did not result in proinflammatory secretory responses by the macrophages (Liu et al., 1999; Heasman et al., 2003). There are several other pathways that are amenable to pharmacological targeting and the multimodal approach, particularly of provoking granulocyte apoptosis in combination with enhancing efferocytosis efficiency is an area of exciting potential.

## Summary

As indicated above, as well as being potential anti-cancer drugs, CDKIs have repeatedly been shown to expediate the resolution process of many inflammatory diseases and injury models by promoting granulocyte apoptosis *in vitro* and *in vivo*. The mechanisms by which apoptosis is induced in granulocytes are consistently highlighted as that of the caspase dependent intrinsic pathway through Mcl-1 reduction and increased mitochondrial membrane potential. CDK9 has also been demonstrated to be the critical target of CDKIs in inducing neutrophil apoptosis. At the CDKI concentrations used in these studies, other non-granulocyte cells are not reported to undergo apoptosis and indeed some other cell types are protected by CDKIs. Efferocytosis, a required component of resolution, particularly with increased cellular apoptosis, is unaffected or in some models, improved. Therefore, CDKI drugs show promise for the treatment of a number of inflammatory conditions by driving granulocyte apoptosis and enhancing inflammation resolution. It should also be noted that in several studies using various preclinical models of inflammatory diseases, CDKI drugs show benefits that may involve other, less defined, anti-inflammatory mechanisms of action (Clough, 2002; Zoja et al., 2007; Sekine et al., 2008; Meijer et al., 2016). The work detailed here indicates an exciting avenue for therapeutic development and opens opportunities for additional studies to establish the breadth of CDKI applications.

## Author Contributions

All authors listed have made a substantial, direct and intellectual contribution to the work, and approved it for publication.

### Conflict of Interest Statement

The authors declare that the research was conducted in the absence of any commercial or financial relationships that could be construed as a potential conflict of interest.
